# Motivation of medical students: selection by motivation or motivation by selection

**DOI:** 10.1186/s12909-016-0560-1

**Published:** 2016-01-29

**Authors:** Anouk Wouters, Gerda Croiset, Francisca Galindo-Garre, Rashmi A. Kusurkar

**Affiliations:** VUmc School of Medical Sciences, Research in Education, PK KTC 5.002, Post box 7057, 1081 BT Amsterdam, The Netherlands; LEARN! Research institute for learning and education, Faculty of Psychology and Education, VU University, Amsterdam, The Netherlands; Department of Epidemiology & Biostatistics, VU University Medical Center, Amsterdam, The Netherlands

**Keywords:** Admission, Motivation, Selection, Self-Determination Theory

## Abstract

**Background:**

Medical schools try to implement selection procedures that will allow them to select the most motivated students for their programs. Though there is a general feeling that selection stimulates student motivation, conclusive evidence for this is lacking. The current study aims to use the perspective of Self-determination Theory (SDT) of motivation as a lens to examine how medical students’ motivation differs in relation to different selection procedures. The hypotheses were that 1) selected students report higher strength and autonomous motivation than non-selected students, and 2) recently selected students report higher strength and autonomous motivation than non-selected students and students who were selected longer ago.

**Methods:**

First- (Y1) and fourth-year (Y4) medical students in the six-year regular programme and first-year students in the four-year graduate entry programme (GE) completed questionnaires measuring motivation strength and type (autonomous-AM, controlled-CM). Scores were compared between students admitted based on selection, lottery or top pre-university GPA (top GPA) using ANCOVAs. Selected students’ answers on open-ended questions were analysed using inductive thematic analysis to identify reasons for changes in motivation.

**Results:**

The response rate was 61.4 % (*n* = 357). Selected students (Y1, Y4 and GE) reported a significantly higher strength of motivation than non-selected students (Y1 and Y4 lottery and top GPA) (*p* < 0.01). Recently selected students (Y1 and GE) reported significantly higher strength (*p* < 0.01) and higher AM (*p* < 0.01) and CM (*p* < 0.05) than non-selected students (lottery and top GPA) and Y4 students who were selected three years ago. Students described that being selected enhanced their motivation as they felt autonomous, competent and that they belonged to a special group. These reported reasons are in alignment with the basic psychological needs described by Self-Determination Theory as important in enhancing autonomous motivation.

**Conclusions:**

A comprehensive selection procedure, compared to less demanding admission procedures, does not seem to yield a student population which stands out in terms of autonomous motivation. The current findings indicate that selection might temporarily enhance students’ motivation. The mechanism through which this occurs seems to be through feelings of autonomy, competence and relatedness inspired by selection.

**Electronic supplementary material:**

The online version of this article (doi:10.1186/s12909-016-0560-1) contains supplementary material, which is available to authorized users.

## Background

Motivation is an important factor in students’ learning and performance [1]. Furthermore, some researchers have speculated that selection for a medical school program might have a positive effect on students’ motivation [1–3]. Despite the recognition of motivation as an important attribute in medical students, and the attempts of medical schools to select the most motivated candidates [4, 5], motivation remains an understudied factor in selection research. In the current study, the quantity and quality of students’ motivation will be studied in relation to different admission procedures.

**Fig. 1:**
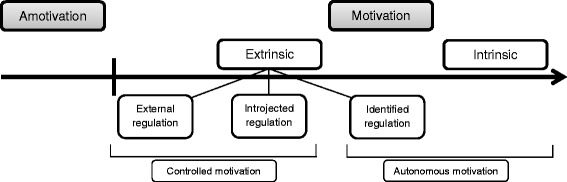
The Self-Determination continuum of motivation (adapted from [13])

Motivation in students has been found to be positively associated with academic performance and learning strategies and negatively associated with dropout behaviour [1]. However, evidence for a direct relationship was not always found and the mechanism is still unknown [1]. Moulaert et al. found positive correlations in their study [6]; whereas, other studies found no significant correlations [2, 7]. Some studies have found that motivation has an indirect relationship with academic performance through deep learning strategy or emotions or resource management [8–10]. Furthermore, research has shown that the quality of motivation is more important for educational outcomes than the quantity of motivation [11]. Self-Determination Theory (SDT) [12] acknowledges the qualitatively different types of motivation and distinguishes between autonomous and controlled types of motivation (see Fig. 1). It will therefore be used as a theoretical framework in this study. Autonomous motivation has been found to be an especially important favourable factor in education. Autonomous motivation concerns intrinsic motivation (doing something out of interest or enjoyment) or the appreciation of certain behaviour as being personally valuable (identified regulation) [13, 14]. An example is a student who is passionate about the functioning of the human body and believes helping others is important. Autonomous motivation has been found to foster deep learning, better study behaviour, higher academic achievement and the intention to continue medical studies; and results in lower dropout rates in (medical) students [1, 8, 9, 14, 15]. Alternatively, controlled motivation implies that behaviour is driven by the promise of reward or the threat of punishment (external regulation), or by internal pressure such as feelings of guilt or shame (introjected regulation). An example is a student who chooses to study medicine in order to please his parents or because of the prospect of a generous salary. A combination of high intrinsic and low controlled motivation in students has been found to demonstrate the most favourable learning behaviours and performance. Unmotivated students and students with a combination of high controlled and low autonomous motivation have shown the least desirable learning behaviours and performance [16].

Medical school selection committees seek the most suitable applicants in terms of performance and motivation [4]. Typically, students who were rejected during the selection process are difficult to recruit for research (as a comparison group for the selected students). Because the Dutch admission system employs different routes for admission, it provides a great opportunity to conduct such comparisons. The three routes are admission based on a pre-university Grade Point Average (GPA) of ≥ 8 on a scale of 1 to 10 (*top GPA*), a weighted lottery for applicants with a GPA below eight (*lottery*), and a qualitative selection procedure (*selection*). If rejected during the selection process, applicants are automatically enrolled in the weighted lottery [17]. In addition, some medical schools offer a graduate entry programme (GE) for which students with a Bachelor’s degree are admitted through a qualitative selection procedure. In some studies, students admitted based on a voluntary qualitative selection procedure have been found to outperform students admitted based on lottery on outcome measures such as dropout, professionalism and study progress [18–21]. Differences in motivation might explain these findings.

Medical schools spend a considerable amount of time and money on the assessment of motivation as part of the selection procedure. This is usually operationalised in the form of personal statements, interviews, multiple mini interviews (MMI’s), etc. [22, 23]. However, measuring motivation during the selection process is challenging because if applicants know their motivation is being measured, they may try to record answers that admissions staff will find favourable (as opposed to their true feelings on their own motivation). A recent study, for example, brought to light that one cannot distinguish between selected and non-selected applicants on the basis of written statements on motivation [24]. Though qualitative selection procedures differ between medical schools, they are all demanding in nature. This might encourage only very motivated candidates to apply. Research on the relationship between selection and motivation is scarce. Some studies have found that selected students have higher strength of motivation than lottery admitted and top GPA students [2, 25], indicating that the most motivated candidates are admitted through selection. However, other studies have not found significant differences [26]. Few studies have examined students’ quality of motivation in relationship to how they were admitted to the medical study. Researchers in a study conducted in the Netherlands found that students admitted through selection reported higher autonomous motivation than lottery students [8].

SDT considers motivation as a factor that influences educational outcomes, as well as a factor that can be influenced by the educational environment [12]. According to SDT, motivation is dynamic and can change from autonomous to controlled and vice versa [13]. When three basic psychological needs—the need for autonomy (the feeling of volition in one’s actions), competence (the feeling of being capable of reaching one’s goals) and relatedness (the sense of belonging)—are fulfilled, students’ intrinsic motivation thrives [13, 27]. Research has shown that educational environment can also influence students’ motivation. For example, a problem-based learning curriculum was found to stimulate students’ intrinsic motivation because it led them to feel like autonomous learners [1]. Researchers have suggested that selection positively affects motivation [1–3]. Moreover, motivation has been reported to decrease throughout the first year of medical school [28]. Following this, recently selected students could be expected to show higher motivation than students who were not recently selected. To our knowledge, this has not yet been explored.

The aim of the current study was to examine the association between selection and motivation. Considering the literature, it is hypothesised that 1) selected students report higher strength and autonomous motivation than non-selected (lottery and top GPA) students, and 2) recently selected students report higher strength and autonomous motivation than students who were selected longer ago and non-selected (lottery and top GPA) students. Our research questions were:

1. Do selected medical students differ from non-selected (lottery and top GPA) students in terms of strength and type of motivation?
2. Do recently selected students, thus students who just entered medical school, differ from non-selected students and fourth-year selected students in terms of strength and type of motivation?
  To examine the mechanism, a third research question was posed.
3. What do selected students report about the influence selection had on their motivation?

These questions were addressed by collecting quantitative data on the motivation of medical students who were admitted through different routes within one medical school and qualitative data on the influence of selection on motivation as perceived by selected students. The Dutch admission system provided a unique research setting, which enabled the comparison of selected students with non-selected students.

## Methods

### Setting

The current study was conducted during the academic year 2012–2013 at VUmc School of Medical Sciences, Amsterdam, which offered two medical tracks (a regular track and a graduate entry track), both with different admission policies. The regular 6-year track consisted of 3 years of pre-clinical education followed by 3 years of clinical education, after which the students received a Medical Degree. For this regular 6-year track, the students were admitted through either a qualitative selection procedure, lottery or based on a top GPA, usually shortly after finishing high school. The selection procedure for the regular track consisted of two steps. In the first step, non-academic attributes were assessed, including the quality and quantity of extracurricular activities (during high school) in health care and management, leadership and organization, and extraordinary achievement in sports, arts or science. Completion of extracurricular courses was also considered relevant. Scores were assigned for relevant activities which were carried out during the 3 years preceding the selection procedure. Provision of evidence for these activities was mandatory. All applicants who met the set minimum score were invited to participate in the second step of the selection procedure. The second step consisted of lectures followed by cognitive tests. Applicants were tested on their study skills and information processing skills using study material of a medical subject. The procedure is similar to the one which has been described in detail elsewhere [18]. The 4-year graduate entry programme in medicine and research (GE) consists of a preparatory year, followed by the regular 3-year clinical education with additional scientific training. GE students already completed 3 years of college education and were admitted based on a three-step qualitative selection procedure, which consisted of a cognitive test, scoring of application forms and MMI’s. This procedure has been described in detail elsewhere [24].

### Study population

The study population consisted of first-year (Y1) and fourth-year (Y4) regular track students, and first-year GE students. All selected students, irrespective of the timing of their selection, comprised the “selection” group, and all lottery and top GPA students comprised the “non-selection” group. The first-year selected students (students selected several months before this study was conducted) comprised the “recently selected” group and all other students comprised the “non-(recently) selected” group. Details on the composition of the groups are provided in Table 1. Sample size calculations, performed using g*power software [29], indicated the need for a total of 269 participants to obtain a medium effect size (Cohen’s *d =* 0.5) [30].

**Table 1:** Group composition for statistical comparisons to test the hypotheses on strength and type of motivation

Hypothesis 1 To study the influence of motivation on selection the strength and type of motivation of the following two groups were compared	
Group 1	Group 2
Selected students	Non-selected students
Y1_selection_ + GE + Y4_selection_	Y1_lottery_ + Y1_top GPA_ + Y4_lottery_ + Y4_top GPA_
Hypothesis 2 To study the influence of selection on motivation the strength and type of motivation of the following two groups were compared	
Group 1	Group 2
Recently selected students	Non-(recently) selected students
Y1_selection_ + GE	Y1_lottery_ + Y1_top GPA_ + Y4_lottery_ + Y4_top GPA_ + Y4_selection_

Hypothesis 1: selected students report higher strength and autonomous motivation than non-selected (lottery and top GPA) students

Hypothesis 2: recently selected students report higher strength and autonomous motivation than students who were selected longer ago and non-selected (lottery and top GPA) students

*Y1*_*selection, lottery, top GPA*_ Year 1 students who are recently admitted based on selection procedure, weighted lottery and top GPA respectively

*Y4*_*selection, lottery, top GPA*_ Year 4 students who are admitted longer ago based on selection procedure, weighted lottery and top GPA respectively

*GE* Graduate Entry students who are recently admitted based on selection procedure

### Procedure

In the first weeks of their Y1, Y4 and GE-Y1 years, respectively, students were invited to participate by filling out a survey with two motivation questionnaires and a few open-ended questions (see Additional file 1). Participants were informed about the research objectives by means of an information letter which stated that the effects of selecting students for the medical study were studied by exploring the motivation of students admitted through different admission procedures.

### Instruments

Strength of motivation, defined as “*students’ readiness to start and continue medical training regardless of sacrifices, setbacks, misfortune or disappointing perspectives*” [26] was measured using the Strength of Motivation for Medical School questionnaire (SMMS-R, Cronbach’s α = 0.79 [31]). The SMMS-R is a 15-item questionnaire using a 5-point Likert scale (1 = completely disagree; 5 = completely agree). Examples of items from the SMMS-R are “*I would still choose medicine even if that meant I would never be able to go on holidays with my friends anymore*” and “*I wouldn’t consider any other profession than becoming a doctor*”.

The type of motivation was measured using the Learning Self‐Regulation Questionnaire (LSRQ) [32] after translating it into Dutch. The LSRQ is a 12-item questionnaire using a 7-point Likert scale (1 = not at all true; 7 = very true). It has two subscales, *Autonomous Motivation*, AM, (seven items) and *Controlled Motivation*, CM (five items), with reported reliabilities of 0.75 and 0.67 for the Cronbach’s α’s, respectively [32]. Examples of AM and CM items are “*The reason that I will work to expand my medical knowledge is…. because it’s interesting to learn more about the nature of medicine*” and “*I will participate actively in the medical courses…. because others might think badly of me if I didn’t*”, respectively.

The open-ended questions were “*Did selection for the medical study have an effect on your study motivation? If yes, how and why?*” and “*Did selection for the medical study have an effect on how you feel about yourself? If yes, how and why?*”. These were constructed, discussed and agreed upon by the research team.

### Analyses

First, the data were screened for accuracy of data entry and missing values, and the variables were checked for normality. Missing values were handled by pairwise deletion. There were nine, six and three missing values for the SMMS-R, AM and CM total scores, respectively. Reliability analyses were carried out for the SMMS-R and the LSRQ subscales. Descriptive statistics and Pearson’s correlations were calculated for all variables. Analyses of covariance (ANCOVA) were carried out to compare the groups mentioned in Table 1. To examine differences in students’ motivation in relationship to admission through a qualitative selection procedure versus admission through lottery and top GPA, strength and type of motivation were compared for the “selection” group and the “non-selection” group. To examine whether first-year selected medical students differed from first- and fourth-year medical students admitted through lottery and top GPA, and fourth year selected students, strength and type of motivation were compared for the “recently selected” group and the “non-(recently) selected” group (i.e. fourth-year selected students and non-selected students). Age and gender were treated as covariates in the analyses because motivation has been found to increase with age [1, 25], and to be higher and more intrinsic in female than male students in some studies [1, 33]. Multiple comparisons were corrected for by performing Bonferroni post-hoc analyses. Cohen’s effect size of difference [30] was calculated for every statistically significant finding.

The selected students’ written answers to the open-ended questions were analysed using thematic analysis [34] in order to identify reasons for change in motivation due to selection. One author (AW) familiarized herself with the data, read the students’ responses iteratively and simultaneously generated and refined categories that were formed by clustering reported reasons. An example is the clustering of the reasons concerned with ‘reflection on study choice’ and ‘getting acquainted with course material’ to form the category ‘informed choice’. Identified reasons and categories were discussed and agreed upon within the research team. A semantic and realist approach was adopted, which means that the categories and meanings were identified from the explicit answers of the students [34].

### Ethical approval

Written informed consent was obtained from all participants. The data were anonymised before analyses. The study was approved by the Ethical Review Board of the Netherlands Association for Medical Education (NVMO-ERB, dossier number 184).

## Results

A total of 357 out of 581 students participated in this research, giving a response rate of 61.4 %. One student was admitted to a medical program based on special circumstances and was, therefore, excluded from the analysis. All (100 %, *n* = 21) GE students, 47.4 % (*n* = 162) of the Y1 students and 80.1 % (*n* = 173) of the Y4 students participated in the study. Of all respondents, 43.8 % (*n* = 156) were admitted through selection, 44.7 % (*n* = 159) were admitted through lottery and 11.5 % (*n* = 41) were admitted because of a top GPA. The average age of the participants was 21.15 years (range = 17 to 41 years), and the gender distribution was representative of that in Dutch medical schools: 28.9 % males and 71.1 % females [17]. The study sample was representative of the study population. The average age of the total study population was 21.38 years (in September 2012), with 69.8 % females. Of the total study population, 43.0 % were admitted through selection, 40.5 % were admitted through lottery and 8.9 % were admitted because of a top GPA (for 7.6 % the way of admission was unknown or because of special circumstances). The descriptives for all groups in the analysis are depicted in Table 2.

**Table 2:** Descriptives (gender, age, motivation scores) and comparisons between groups using ANCOVA with age and gender as covariates

	Female (%)	Mean age ± SD	Strength of motivation for medical school, Mean^a^ (SE)	Autonomous Motivation, Mean^a^ (SE)	Controlled Motivation, Mean^a^ (SE)
			(min. score = 15, max. score = 75)	(min. score = 7, max. score = 49)	(min. score = 5, max. score = 35)
Hypothesis 1					
Selected students (*n* = 156)	71.8 %	21.33 ± 3.01	54.84 (0.66)	5.99 (0.05)	4.14 (0.07)
Non-selected students (*n* = 200)	70.5 %	21.01 ± 2.63	52.28 (0.58)	5.86 (0.05)	4.15 (0.06)
Test Value			*F* = 8.516**	*F* = 3.470 (n.s.)	*F* = 0.012 (n.s.)
Effect size (Cohen’s *d*)			*d* = 0.32	*d* = 0.19	*d* = 0.04
Hypothesis 2					
Recently selected students (*n* = 90)	77.8 %	19.80 ± 2.13	56.77 (0.88)	6.12 (0.07)	4.31 (0.09)
Non-(recently) selected students (*n* = 183)	68.8 %	21.61 ± 2.86	52.27 (0.50)	5.85 (0.04)	4.09 (0.05)
Test Value			*F* = 19.146**	*F* = 11.032**	*F* = 4.421*
Effect size (Cohen’s *d*)			*d* = 0.50	*d* = 0.48	*d* = 0.44

Hypothesis 1: selected students report higher strength and autonomous motivation than non-selected (lottery and top GPA) students

Hypothesis 2: recently selected students report higher strength and autonomous motivation than students who were selected longer ago and non-selected (lottery and top GPA) students

Effect size values of 0.2, 0.5 and 0.8 are considered small, medium and large respectively

*SD* standard deviation; *SE* standard error

*p < .05

**p < .01

^a^Adjusted for covariates age and gender

The Cronbach’s alpha values for reliability were 0.79, 0.63 and 0.62 for the SMMS-R, Autonomous Motivation and Controlled Motivation, respectively.

The distribution of the scores was broadly normal, with the exception of a moderate negative skewness for the Autonomous Motivation scores. This was not expected to cause inferential problems because ANCOVA has been found to be robust to moderate violations of the normality assumption [35]. Table 3 depicts the correlations among strength of motivation, autonomous motivation and controlled motivation. Significant correlations were found between all variables. The correlation between strength of motivation and autonomous motivation is in line with other findings in the literature [31].

**Table 3:** Pearson’s correlations among variables

Variable	1	2	3
1. Strength of motivation for medical school	—		
2. Autonomous Motivation	0.388^a^	—	
3. Controlled Motivation	0.132^b^	0.256^a^	—

^a^Correlation is significant at the 0.01 level (2-tailed)

^b^Correlation is significant at the 0.05 level (2-tailed)

The quantitative results for the first two research questions are depicted in Table 2. Scores on both autonomous and controlled motivation found in the current study are similar, or slightly higher [32, 36, 37], in comparison with other studies using the LSRQ. The average scores on strength of motivation were comparable with those found in other studies using the SMMS [2, 7, 25, 26, 38].

### Selected students compared with non-selected students

Strength of motivation for medical school was significantly higher for the “selection” group in comparison with the “non-selection” group (*F* = 8.516, *p* = 0.006, Cohen’s *d* = 0.32, effect size small to medium). No statistically significant differences were found between the “selection” group and the “non-selection” group regarding autonomous (*F* = 3.470, *p* = 0.063) and controlled motivation (*F* = 0.012, *p* = 0.912).

### Recently selected students compared with fourth-year selected students and non-selected students

Strength of motivation for medical school (*F* = 19.146, *p* = 0.000, Cohen’s *d* = 0.50, effect size medium), autonomous motivation (*F* = 11.032, *p* = 0.000, Cohen’s *d* = 0.48, effect size small to medium), and controlled motivation (*F* = 4.421, *p* = 0.001, Cohen’s *d* = 0.44, effect size small to medium) were significantly higher for the “recently selected” group in comparison with the “non-(recently) selected” group.

Additional ANCOVAs, comparing only selected students (i.e. Y1_selection_, Y4_selection_ and GE-Y1), revealed the same pattern. Strength of motivation for medical school (*F* = 18.720, *p* = 0.000, Cohen’s *d* = 0.47, effect size small to medium), autonomous motivation (*F* = 12.248, *p* = 0.001, Cohen’s *d* = 0.62, effect size medium to large), and controlled motivation (*F* = 6.647, *p* = 0.011, Cohen’s *d* = 0.84, effect size large) were significantly higher for Y1_selection_ students and GE-Y1 students in comparison with the Y4_selection_ students. The effect sizes for the differences in autonomous and controlled motivation increased when the recently selected students were compared with only the students who were selected 3 years ago.

Students (*n* = 134) reported that their motivation had increased due to selection (scores 5 to 7 on a scale of 7). Some students (*n* = 7) reported that their motivation had not changed at all (a score of 1 on a scale of 7), mainly because they stated that they were already very motivated. Eleven of the selected students did not provide answers to the open-ended questions. Table 4 shows responses and quotations of the students on their perceived reasons for change in their motivation due to selection, arranged by the categories identified during the analysis. These categories were in alignment with the three psychological needs described by SDT, i.e. *autonomy*, *competence* and *relatedness*. Additional categories that were identified were *informed choice* and *result of effort*.

**Table 4:** Reasons for change in motivation due to selection as reported by students, illustrated with quotations

Reasons for change in motivation	Quotations
Autonomy	
Feeling in control	*“Selection made you feel like you were in control of your admission, which was very nice”* (this presumably is in comparison with weighted lottery)
Competence	
Affirmation of their abilities	
- By themselves	*“It gave me confidence that I can handle it, that I am fit for the programme”*
- By others	*“… because selection confirmed that others also considered me suitable”*
- Living up to expectations	*“A better/extra chance to prove to myself that I can do it”*
Relatedness	
Feeling privileged	
- Being part of a special group	*“By being part of a special group” & “Because being part of a group of lucky people is an honour”*
- Getting a chance	*“Because I got the chance to study what I always wanted”*
Informed choice	
Reflection on study choice	*“Because the selection is tough, you reflect on why you want this so badly”*
	*“… and you are constantly asked about your motivation by your environment and by yourself”*
Getting acquainted with course material	*“During selection you could already get a taste of the study, which (luckily) felt good”*
Result of effort	*“Because of the dozens of extra hours I put into my preparation for decentralized selection I am more motivated. You put in more ‘effort’ in order to be able to study this, therefore you want to go for it even more!”*

## Discussion

The main purpose of this study was to examine the association between selection and motivation. The results indicate that selected students are more motivated, but do not show different types of motivation compared to non-selected students. Furthermore, the results seem to support the hypothesis that selection stimulates students’ motivation, but also indicate that this might last only for a short period of time. Selected students reported higher strength of motivation than non-selected students, which is in line with findings from other research [2, 25]. A reason for this finding in our institute could be the demanding nature of the selection procedure for which time investment in healthcare activities is a criterion. This might result in the most motivated students applying and succeeding in selection, as has previously been suggested by other researchers [20]. Research on differences in type of motivation between selected and non-selected students is scarce. Kusurkar et al. found higher autonomous motivation in selected students [8]. The sample size of selected students in the Kusurkar et al. study was very small. We could not replicate this finding in a larger sample.

Furthermore, this study gives a first indication of a positive, though possibly only temporary, effect of selection on motivation. Such an effect has been hypothesized before [1–3], but to our knowledge, this is the first study which attempts to address this issue. Students who were recently selected reported higher strength and autonomous and controlled types of motivation, which suggests that the presence of a selection procedure might enhance the quantity and quality of students’ motivation for medical school. Enhanced motivation was also reflected in selected students’ answers to the open-ended questions. An explanation for the higher autonomous motivation reported by recently selected students can be sought in the fulfilment of the students’ needs for autonomy (being in control of their admission), competence (feeling able to handle the programme) and relatedness (being part of a special group). The reasons provided by the students also helped explain the enhancement of controlled motivation. Examples are that the selection committee (thus an external factor) considered the students to be fit for medical school, which made the students want to prove themselves to others, and continuously being questioned about their motivation by people in their environment. Remarkably, some students described that selection enhanced their motivation only during the first few weeks of their studies. If selection has an enhancing effect on motivation, it might be of temporary nature, followed by a decrease of motivation during the first year of medical school, as was found in a longitudinal study [28]. We may have found an indication for a possible temporary nature of an enhancing effect because of the unique Dutch setting. At first, selected students might feel special and privileged compared to students who were admitted based on other, less demanding, procedures. As more time since selection passes, however, the educational system might have a larger influence on their motivation. Due to government policy, the Dutch admission system is moving from three different procedures to selection-only. It would be interesting to investigate whether the current findings can be generalised to a selection-only situation. Selection might still address students’ needs for autonomy, competence and relatedness, but possibly to a lesser extent, because their peers will all be admitted through the same procedure.

Some important limitations should be considered when interpreting the results from this study. The most important limitation is that this is not a longitudinal, but a cross-sectional study. To study the effect of selection on motivation, a longitudinal study would be most desirable. However, the assessment of motivation in a high stakes situation, such as selection, is likely to generate social desirable answers [24, 39], which hampers a proper pre- and post-selection comparison. In the current study, we tried to gain insight into the underlying mechanisms by combining qualitative and quantitative data. Although the combined results give an indication that such an effect might exist, a more direct method to examine this effect is desirable. We are planning a longitudinal study to explore the evolution of students’ motivation through their medical study in relationship to the different admission procedures. A second limitation is the Cronbach’s α for the LSRQ, which was just below the desirable value of 0.7. However, because the comparisons were at group level and the overall sample size was good, we found it acceptable. Finally, the study was conducted at one single university in the Netherlands, limiting the generalizability of the findings. More studies on the association between selection and motivation, in different universities and settings, are recommended.

Considering the motivation scores reported in our study, students are already motivated when they enter medical school. In addition, students admitted through a demanding selection procedure experience an increase in motivation in the first period of their medical course. In order to retain all students’ motivation throughout the medical curriculum, especially autonomous motivation (which is associated with deep learning strategies, high study efforts and ultimately higher academic achievement [8]), the learning environment could be arranged in such a way that the students’ needs for autonomy, competence and relatedness are satisfied. This can be realised by offering autonomy-supportive education [3, 40, 41]. Problem-based learning curricula, blended learning, early contact with and responsibility for patients, standards-based assessment and the opportunity to follow elective courses have been identified as beneficial for students’ motivation [1, 42].

## Conclusions

This study provides insight into the association between selection and motivation. A comprehensive selection procedure, compared to less demanding admission procedures, does not seem to yield a student population which stands out in terms of autonomous motivation. The current findings indicate that a temporary enhancing effect of selection on motivation might exist, but a more reliable way to study the effect of selection on motivation is necessary. Efforts could be undertaken by medical schools to preserve the students’ autonomous motivation by offering autonomy supportive education.

## Additional file

Additional file 1:
**Survey used in the study (English version).** PDF 63 kb)

## Abbreviations

AM: Autonomous motivation
CM: Controlled motivation
GE: Graduate entry
GPA: Grade point average
MMI: Multiple Mini Interview
SDT: Self-Determination Theory
Y1: Year 1
Y4: Year 4
