# Supplementary material for: Motivation of medical students: selection by motivation or motivation by selection
Source: BMC Med Educ. 2016 Jan 29;16:37. doi: 10.1186/s12909-016-0560-1 (PMC4731894; doi:10.1186/s12909-016-0560-1)
Supplement: Additional file 1: — Survey used in the study (English version). PDF 63 kb) [file 12909_2016_560_MOESM1_ESM.pdf]

Additional file 1: Survey used in the study (English version)

Name*: ………………………………………………………………………………

Gender: 0 Male 0 Female

Age: ………. years

High School GPA: ……........

* Your name will be replaced by a number in order to ensure anonymous handling of your data.

---

1) People have diverse reasons to study medicine. Please indicate how much the following statements reflect your personal situation by marking 1 to 5 (strongly disagree to strongly agree) with each statement below.

**1** strongly disagree — 2 — **3** — 4 — **5** strongly agree	
1. I would always regret my decision if I hadn't availed myself of the opportunity to study medicine.	1 – 2 – 3 – 4 – 5
2. I would quit studying medicine if I were 95% certain that I could never become the specialist of my choice.	1 – 2 – 3 – 4 – 5
3. I would still choose medicine even if that would mean studying in a foreign country in a language that I have not yet mastered.	1 – 2 – 3 – 4 – 5
4. As soon as I would discover that it would take me ten years to qualify as a doctor, I would stop studying.	1 – 2 – 3 – 4 – 5
5. Even if I could hardly maintain my social life, I would still continue medical training.	1 – 2 – 3 – 4 – 5
6. I wouldn't consider any other profession than becoming a doctor.	1 – 2 – 3 – 4 – 5
7. I would still choose medicine even if that meant I would never be able to go on holidays with my friends anymore.	1 – 2 – 3 – 4 – 5
8. I would stop studying medicine if I started scoring low marks and failing tests often.	1 – 2 – 3 – 4 – 5
9. If studying took me more than an average of 60 hours a week, I would seriously consider	1 – 2 – 3 – 4 – 5

quitting.	
10. I intend to become a doctor even though that would mean taking CME courses two evenings a week throughout my professional career.	1 – 2 – 3 – 4 – 5
11. It wouldn't really bother me too much if I could no longer study medicine.	1 – 2 – 3 – 4 – 5
12. I would like to become a doctor, even if that would mean giving precedence to my work over my family.	1 – 2 – 3 – 4 – 5
13. I would quit studying as soon as it became apparent that there were no jobs or resident positions after graduation.	1 – 2 – 3 – 4 – 5
14. I would not have chosen medicine if it would have caused me to accumulate substantial financial debts.	1 – 2 – 3 – 4 – 5
15. I would be prepared to retake my final high school exams to get higher marks if this would be necessary to study medicine.	1 – 2 – 3 – 4 – 5

**1**	2	**3**	4	**5**
strongly disagree				strongly agree

2) The following questions relate to your reasons for participating actively in the courses. Different people have different reasons for their participation in their studies, and we want to know how true each of the reasons is for you. Please indicate how true each reason is for you below.

**1**	2	3	**4**	5	6	**7**
not at all true			somewhat true			very true

A. I will participate actively in the medical courses:	
1. Because I feel like it's a good way to improve my understanding of the material.	1–2–3–4–5–6–7
2. Because others might think badly of me if I didn't.	1–2–3–4–5–6–7
3. Because I would feel proud of myself if I did well in my studies.	1–2–3–4–5–6–7
4. Because a solid understanding of medicine is important to my intellectual growth.	1–2–3–4–5–6–7
B. I am likely to follow my teachers' suggestions for studying:	
5. Because I would get a bad grade if I didn't do what they suggest.	1–2–3–4–5–6–7
6. Because I am worried that I am not going to perform well in my studies.	1–2–3–4–5–6–7
7. Because it's easier to follow their suggestions than come up with my own study strategies.	1–2–3–4–5–6–7
8. Because they seem to have insight about how best to learn the material.	1–2–3–4–5–6–7
C. The reason that I will work to expand my knowledge of medicine is:	
9. Because it's interesting to learn more about the nature of medicine.	1–2–3–4–5–6–7

10. Because it's a challenge to really understand how to solve medical issues.<br>11. Because good grades for the medical courses will look positive on my record.<br>12. Because I want others to see that I am intelligent.	1–2–3–4–5–6–7<br>1–2–3–4–5–6–7<br>1–2–3–4–5–6–7

**1**	2	3	**4**	5	6	**7**
not at all true			somewhat true			very true

3) How were you admitted to the medical programme at VUmc?

0 Through lottery for the regular track (proceed to question 4)

0 Direct admission to the regular track because of a high school GPA of 8 or higher (proceed to question 5)

0 Through selection for the regular track (proceed to question 7)

0 Through selection for the graduate entry track (proceed to question 7)

4) Have you participated in a selection procedure for the medical study prior to admission through lottery?

0 Yes, selection for the regular track

0 Yes, selection for the graduate entry track

0 No

5) Did admission to the medical study have an effect on your study motivation? Indicate below.

**1**	2	3	**4**	5	6	**7**
not at all true			somewhat true			very true

Open questions:

<u>How</u> did admission to the medical study have an effect on your study motivation?

<u>Why</u> did admission to the medical study have an effect on your study motivation?

6) Did admission to the medical study have an effect on how you feel about yourself? Indicate below.

**1**	2	3	**4**	5	6	**7**
not at all true			somewhat true			very true

Open questions:

<u>How</u> did admission to the medical study have an effect on how you feel about yourself?

<u>Why</u> did admission to the medical study have an effect on how you feel about yourself?

This was the last question. Thank you for your participation.

7) Did selection for the medical study have an effect on your study motivation? Indicate below.

**1**	2	3	**4**	5	6	**7**
not at all true			somewhat true			very true

Open questions:

How did selection for the medical study have an effect on your study motivation?

Why did selection for the medical study have an effect on your study motivation?

8) Did selection for the medical study have an effect on how you feel about yourself? Indicate below.

**1**	2	3	**4**	5	6	**7**
not at all true			somewhat true			very true

Open questions:

How did selection for the medical study have an effect on how you feel about yourself?

Why did selection for the medical study have an effect on how you feel about yourself?

This was the last question. Thank you for your participation.
